# Prevention and Treatment of Hepatitis Virus Infections in Hematopoietic Stem Cell Transplant Recipients

**DOI:** 10.4084/MJHID.2009.017

**Published:** 2009-12-10

**Authors:** Suparno Chakrabarti, Somnath Mukherjee

**Affiliations:** Manashi Chakrabarti Foundation For Blood Disorders, Kolkata- 700032, INDIA

## Abstract

Infections with Hepatitis viruses B and C pose major problems both short and long term respectively after HSCT. The key to prevention for Hepatitis B disease remains vaccination for HBV-naïve patients and judicial use of anti-viral therapy in both pre- and post-transplant settings for HBV-infected patients. HBsAg positive grafts to HBV-naïve recipients result in transmission of the virus in about 50%. The newer anti-viral agents have enabled effective treatment of post-transplant patients who might be lamivudine-resistant or might develop so. Selecting a previously infected donor who has high titres of surface antibody for HBsAg positive patients gives the best chance for immunological clearance. The most challenging aspect of preventing HBV reactivation remains the duration of anti-viral therapy and timing of its withdrawal as most reactivations and often fatal ones occur after this period. Hepatitis C, on the other hand affects long-term survival with early onset of fibrosis and cirrhosis. Early effect of Hepatitis C virus on the immune system remains conjectural. The standard combination therapy seems to be effective, but data on this front remains sparse, as in the case of the use of newer antiviral agents. HSCT from HCV infected grafts result in more consistent transmission of the virus and pre-donation treatment of donors should be undertaken to render them non-viremic, if possible. The current understanding and recommendations regarding prevention and management of these infections in HSCT recipients are discussed.

## Introduction:

Haematopoietic Stem Cell Transplantation (HSCT) has evolved over the last three decades from a procedure limited to high-risk haematological malignancies with high risk of morbidity and mortality to a much more widely applicable one across age and donor barriers. Given the explosive expansion of indications for HSCT and the new and novel techniques of transplantation, the nature of complications, particularly the opportunistic infections have evolved as well. Hepatitis B and C virus infections are widely prevalent amongst the general population, though differentially across the various continents. The impact of these infections on transplant outcome is getting better recognised. A recent guideline published jointly by the CIBMTR, CDSC and other organisations have tried to address these issues from a global perspective^1^. In this article we shall discuss the issues related to the prevention and treatment of Hepatitis B and C in patients receiving HSCT. The recommendations made are categorised as per the level of evidence (Table 1).

## HEPATITIS B

### Epidemiology:

Over 350 million people are HBV carriers worldwide, with the infection being endemic in several countries. The prevalence of chronic hepatitis B infection in endemic areas range from 5–25%, whereas, that in non-endemic regions are less than 0.5%^2^,^3^. The prevalence of HBsAg positivity in patients with haematological malignancies in the USA and western Europe is between is between 1–3.5%. In endemic populations in Asia, the frequency of the same goes up to 10%^4^,^2^,^3^.

### Natural history of hepatitis B infection:

The course and outcome of HBV infection depends on two factors: age at infection and the host-virus interaction. Neonatal infection usually results in persistent infection, but acquisition of the virus later in life most commonly result in acute hepatitis. Whilst acute viral hepatitis is self-limiting, a small minority (<5%) can progress to fulminant hepatic failure. About 10% of those infected after the first two years of life would be a chronic carrier, with 20% of those developing cirrhosis. Again, 25% of those with hepatitis B associated cirrhosis could develop hepatocellular carcinoma^5^(Table 2).

### Risk factors for HBV reactivation post-transplant and long term outcome:

HBV reactivation post-transplant is characterised by rising levels of HBV DNA in blood during the period of severe immunosuppression preceeding the rise in liver enzymes (ALT and AST) at the onset of immune recovery. Unless serially monitored, this sequence of events might be missed, as the HBV load might not be as high at the onset of clinical hepatitis which marks the recovery of cellular immunity. Although any chemotherapeutic or immunosuppressive agent used during conditioning increases the risk of HBV reactivation, steroids pose the greatest risk. Steroids facilitate replication of HBV through a glucocortcoid responsive element in the viral DNA^6^,^7^. Other immunosuppressive agents such as rituximab^8^ and alemtuzumab^9^,^6^,^7^, which are used as a part of conditioning regimen with increased frequency are also associated with increased risk of HBV reactivation. Reduced Intensity conditioning is associated with similar or higher incidence of viral reactivations depending on the use of in-vivo anti-T cell antibodies. There is limited data on Hepatitis B reactivation in this setting and deaths have been reported following cessation of anti-viral therapy. The greatest risk of HBV hepatitis is at the time of withdrawal of immunosuppression and very close monitoring is warranted at that point. Preemptive therapy should cover and extend well beyond this period to negate this effect. Markers of humoral immunity are not reliable indicators of virus specific immunity in the post-transplant period, particularly in those receiving IVIG. Hence, it is recommended that the HBV status be monitored by the viral load. HBeAg and anti-HBe which are often monitored in patients with chronic hepatitis should not be considered as only indicators of viral replication either pre- or post-HSCT. Sensitive and quantitative PCR assays should be employed in diagnosing and monitoring HBV infection pre and post-HSCT. Moreover, patients who have apparently recovered from HBV infection based on serological markers, can still harbour the virus in the hepatocytes^10^. Subsequent to the loss of protective anti-HBs titres and compromised cellular immunity, these patients can reactivate HBV from this site. Hence, in patients with previous infection with HBV, even if the pre-transplant parameters indicated resolved infection, HBV reactivation can still occur from latent sites such as hepatocytes on the loss of protective immunity. This is referred to as *reverse seroconversion*. In the context of autologous transplants, this risk was deemed to be greatest in patients transplanted for myeloma^48^, while in allogeneic transplantation those on high dose steroids and with chronic GVHD are possibly at a greater risk. Precore mutatants (1896 G-A) are associated with a higher risk of HBV reactivation and severe hepatitis^48^. A serological profile of positive HBsAg, positive HBV DNA, positive anti-HBe and negative HBeAg should suggest the presence of pre-core mutataion. This can be confirmed by PCR assay for the mutation or direct sequencing. Early or unexplained breakthrough on lamivudine therapy should also raise suspicion of this mutant form. In addition, superinfection with other hepatotrophic viruses might result in precipitation or worsening of hepatitis in a HBsAg positive recipient^12^. Drugs and GVHD could also complicate the picture. A liver biopsy is warranted in all such situations to resolve the issue, particularly if GVHD is considered. Hepatitis is often a late feature post-allogeneic transplantation with a cumulative incidence of 20% at 5 years for HBsAg positive recipients and 1% in anti-HBs positive recipients^13^. This is noted much earlier in autologous setting. The risk of HBV hepatitis in autologous transplantation is similar to that following an allograft^14^. The risk of hepatitis is reduced only in those achieving sustained clearance of HBV through adoptive transfer of natural immunity from the donor^13^. Unlike HCV, the incidence of cirrhosis or HCC is not increased following allogeneic HSCT in HBsAg positive donors when compared to the normal population^13^.

### HSCT Recipient:

Every HSCT recipient should be screened for evidence of past or current HBV infection. The screening should comprise of anti-HBc, HBsAg and anti-HBs (AII). This should be carried out in every patient much before HSCT, ie before initiation of chemotherapy for malignancy or transfusion for non-malignant disorders or at the earliest stages of consideration for HSCT.

### No past exposure to HBV infection:

If the above parameters are negative, the patient should be immunised with two doses of hepatitis B vaccine at 3–4 weeks interval before initiating chemotherapy or transfusion and preferably the third dose at 6 months (AII). This is often not possible in patients undergoing chemotherapy for newly diagnosed cancers. The third dose in these patients should be administered a few months after completion of chemotherapy. The chances of response to immunisation are over 90% in normal individuals, but this is much lower in patients undergoing chemotherapy (less than 60%).

### Past exposure to HBV infection (AIII):

Past exposure to HBV in a HSCT recipient increases the risk of HBV reactivation post-transplant depending on the sero-status. The possible scenarios are described in Table 3. A negative HBsAg with evidence of antibodies to HBc indicates past infection and does not rule out the possibility of reactivation during post-transplant immunosuppression, unless anti-HBs titres are protective (> 10 IU/l). Hence all such patients with absent or low titres of anti-HBs, should be checked for HBV-DNA. If HBV-DNA is negative, attempt should be made to boost the HBV-immunity with vaccination. Those who fail to achieve protective titres should be monitored closely as they are at high risk of reactivation. All HBsAg positive or HBV DNA positive patients with evidence of recent hepatocellular injury should undergo a liver biopsy prior to HSCT, because pre-existent cirrhosis and hepatic fibrosis can increase transplant-related morbidity and mortality (BIII). It is not recommended routinely outside this setting.

### HSCT Donor:

Donors should be routinely screened using same parameters as the recipient. The possible scenarios with donor-recipient sero-status are detailed in Table 4. This is not an absolute contraindication for selecting a HBsAg positive donor for a non-exposed or HBV-naïve recipient (BIII). The overriding concern must be the HLA-matching and other outcome-related concerns. The risk of transmission to HBV-naïve recipient is not 100% and the exact risk remains unclear, although this can be estimated to be around 50%, based on the current evidence (AII)^15^. Every attempt should be made if possible to immunise the recipient and render the donor non-viremic and the graft in-turn HBV-DNA negative. Those who mount a good antibody response post-vaccination and receive a HBV-negative graft following treatment of the donor are at no greater risk of HBV reactivation than those receiving grafts from HBV-naïve donors. However, in most situations, HSCT could be deemed necessary at the earliest and vaccinating the recipient or treating the donor to non-viremic stage might not be feasible. The best option then lies in reducing the volume of the graft (ie reducing the viral load) and passive immunisation of the HSCT recipient immediate pre-transplant. Such patients should be treated in the same way as HBV infected patients with close monitoring of viral DNA load, liver enzymes and preemptive antiviral therapy. HSCT from HBsAg negative, anti-HBc positive donors depend on their anti-HBs titres and presence or absence of viremia (Table 5). However those previously infected donors who have protective anti-HBs titres should be preferred for a HBV infected patient as this offers the possibility of immunological clearance of HBV following HSCT (see below).

### Preventing HBV reactivation post-transplant:

All patients who are positive for either HBsAg or HBV-DNA and those who receive grafts from similar donors are at the highest risk of HBV reactivation (defined as ‘High Risk’). Those who are negative for HBsAg or HBV-DNA pre-transplant but have low anti-HBs titres or those receiving grafts from HBsAg positive donors who are rendered non-viremic following antiviral therapy are deemed to be at risk of HBV-reactivation albeit lower (defined as ‘Low Risk’). Such patients should be closely monitored for viral load and liver function (Table 6).

There are two approaches to address this.

1. Intervening on diagnosis of hepatitis B reactivation
2. Pre-emptive intervention pre-transplant.

The current evidence weighs heavily in favour of preemptive intervention (AI). In patients who go to autologous HSCT with high viral load (> 10^5^ copies/ml) are at the greatest risk of HBV reactivation^16^. A significant body of evidence including a couple of randomised studies and recent systematic reviews and metanalysis on the role of preemptive intervention in patients undergoing chemotherapy for malignancies strongly support the use of a pre-emptive approach^17^,^18^,^14^,^19^,^20^,^21^. This approach not only reduced the incidence of HBV reactivation and hepatitis, both severe and non-severe, but also significantly reduced both overall mortality and hepatitis associated mortality. Extrapolation of these evidences to HSCT, coupled with several large case-series would justify preemptive approach in these categories.

### Pre-emptive Intervention:

Anti-viral agents which inhibit DNA polymerase are extremely effective in rapidly suppressing viral replication. All the studies referred to above have employed lamivudine at a dose of 100 mg/day. Although lamivudine is relatively non-toxic, prolonged use leads to selection of YMDD mutant strains^2^. Such mutants are generally responsive to adefovir dipivoxil^23^,^24^ or entecavir^25^. In trials on patients with chronic hepatitis, only 3.2% of lamivudine-resistant patients developed resistance to adefovir at 12 months. Moreover, a combination of adefovir and lamivudine led to quicker response and less resistance than adefovir used alone^26^. Newer drugs such as entecavir and tenofovir are more potent anti-virals and the latter is effective against both lamivudine and adefovir-resistant strains^27^. However, there is no study to support their usage as first-line therapy in post-transplant patients, but might be considered in situations where prolonged anti-viral therapy is warranted.

### Duration of therapy:

There is no clear guideline on the optimum duration of treatment. In patients receiving chemotherapy, discontinuation of therapy very soon after completion of chemotherapy leads to HBV reactivation and hepatitis similar to those not receiving treatment. Hence, it is justified to continue antiviral therapy for at least 6 months post-transplant in autologous transplants and 12 months post-transplant in allogeneic transplants, or longer if patient is on immunosuppression for chronic GVHD (BIII). Extended use of lamivudine, not only results in reduction of HBV reactivation and the resultant complications, but also impacts HBV-associated mortality^28^,^29^. Monitoring of HBV-DNA levels and liver enzymes for at-least three months is advisable after stoppage of anti-viral therapy as there is a risk of rebound viral replication and immune-mediated liver injury after withdrawal of both anti-viral and immunosuppressive drugs.

### Adoptive transfer of immunity to HBV:

Immunity to HBV can be adoptively transferred from the donor to the recipient. Anti-HBs positive marrow donation has resulted in durable anti-HBs expression in the recipient^30^. This has also resulted from DLI from vaccinated donors^31^. More importantly, transplants from HBV immune donors to HBsAg positive patients have resulted in sustained clearance of HBV infection. This is more likely to happen after natural infection than after active immunisation^32^. In the largest follow-up study reported to date, 65% of HBsAg positive recipients had sustained clearance of HBsAg, following transplantation from donors with natural immunity to HBV. This process was augmented by preemptive lamivudine therapy^13^.

### Reverse Seroconversion post-transplant:

Patients who are anti-HBs and anti-HBc positive pre-transplant are considered to be at negligible risk of HBV reactivation. However, over 50 cases of reverse seroconversion have been reported in the literature, following both autologous and allogeneic transplantation, with an approximate overall risk of 1%^33^,^13^,^34^. This probably results from undetectable levels of virus in serum or virus residing in hepatocytes with serum negative for HBV DNA. This phenomenon is characterised by progressive loss of anti-HBs antibodies along with rising viral load and reappearance of HBsAg. This can result in asymptomatic carrier state, symptomatic hepatitis or rarely fulminant hepatic failure. Anti-HBs positive patients should have anti-HBs monitored every 3 months and sudden or progressive reduction in anti-HBs titre should prompt HBV DNA test^35^. Patients on high dose steroid therapy or on immunosuppression for chronic GVHD, who receive the graft from a HBV-naïve donor are at particular risk of reverse seroconversion. Those loosing anti-HBs response should receive active immunisation in an attempt to restore protective levels of anti-HBs, if there is no evidence of HBV DNA in serum (BIII).

### Transfusion-Risk:

In UK for example, the risk of acquiring hepatitis B through transfusion is I in 150,000. The same is not true for many Asian or Latin American countries, where the disease is endemic and prevalent in upto 25% of the population in certain areas. The importance of active immunisation in unexposed patients before administration of blood products cannot be overemphasised (AII). However, this is not always practicable and a proportion of patients would be poor-responders. All patients should have the serological profile repeated prior to HSCT, even if they were HBV-naïve and immunised prior to chemotherapy. This is because of the possibility of reverse seroconversion through the period of cytotoxic and immunosuppressive therapy which most would be exposed to prior to transplantation (AIII).

### Sexual Transmission:

Anti-HBs negative HSCT recipients who have recived a graft from HBV-naïve donor and have not been immunised or have failed to mount a response to vaccination are at increased risk of acquiring HBV from an infected partner. Those who experience loss of anti-HBs post-transplant are also at similar risk. Late hepatitis from sexual transmission has been reported^36^ Both groups should be advised adequate sexual protection and early vaccination if the partners are known HBsAg carriers (AIII).

## HEPATITIS C

### Epidemiology:

Globally, about 170 million people are chronically infected with Hepatitis C virus (HCV) and about 3–4 million more are infected each year. The major mode of transmission is via infected blood products and infected needles shared by drug-users or percutaneous procedures. The prevalence of chronic HCV infection is higher in Africa and South East Asia than in Western Europe and North America. However, the disparity is not as pronounced as in HBV, largely because of the millions infected in these places through blood transfusion before 1992, when second generation anti-HCV testing came into being (WHO factsheet). Unsafe blood products still contribute to majority on newly acquired HCV in countries with high prevalence of the infection.

### Natural course and relevance to HSCT:

Majority of patients infected with hepatitis C do not have acute clinical manifestations, unlike HBV. However, 80% of those infected develop chronic infection and 20% of chronic HCV infected cohort develop cirrhosis of the liver after 20–30 years with 1–5% developing HCC. Prior to the identification of hepatitis C in 1989 and effective screening in 1992 onwards, several million people were infected globally through blood transfusion. In a look-back study, Seattle identified that 32% of 355 patients who received a transplant in 1987–88 were infected with HCV^37^. Several studies since have assessed the short and long term consequences of HCV infection in HSCT recipients. There was no evidence of adverse effect of HCV short-term apart from reports of Veno-occlusive Disease (VOD) in some studies. However, it was not until 2004, that the long-term adverse effect of HCV started becoming apparent. Earlier studies of 5–10 years follow-up, did not find any effect of HCV on mortality or morbidity^38^. However, all the studies reported a rise in liver enzymes in HCV positive patients on withdrawal of immunosuppression, but liver failure was extremely rare. Fifteen years follow-up on a cohort of 96 HCV infected patients, revealed a cumulative incidence of biopsy-proven cirrhosis of 11% and 24% at 15 and 20 years respectively^39^. The risk factors for progression to cirrhosis in HCV infected HSCT recipients were extra-hepatic manifestations and genotype C. It was further documented in this study that allogeneic HSCT is the only risk factor for early progression to cirrhosis, occurring at a median of 18 years compared to those not receiving HSCT. The seattle group also reported HCV as the major cause of cirrhosis following allogeneic transplantation^40^. A recent retrospective study on 31 HCV infected patients showed an increase in non-relapse mortality in these patients compared to matched controls. The causes of death were related not to liver disease per se, but more infectious complications. Whether this is secondary to a direct or indirect effect of Hepatitis C virus on the recovering immune system, remains open to speculation. Similar to Hepatitis B, there is scant data on the outcome of Hepatitis C infected patients following reduced intensity transplantation.

## Preventing HCV Infection

### HSCT recipients:

HCV infection in the recipient without any evidence of liver damage is not a contraindication for HSCT. The only short term risk is probably associated with VOD. However, all HSCT recipients should be assessed with careful history, examination and liver function test for the risk of HCV infection (BIII). Anti-HCV antibodies must be tested in all HSCT recipients (AII). Those with history of transfusion before 1992 (this may vary according to the country of origin), IV or inhaled drug abuse, tattoos and unexplained rise in liver enzymes, should undergo nucleic acid testing for HCV despite a negative anti-HCV (AII). Anti-HCV response takes 6 months to be detected or may never be detectable in a small proportion of individuals or during or immediately after prolonged and heavy chemotherapy or immunosuppression^41^. Thus, confirmation of HCV infection is dependent on detection of HCV-RNA. All patients with HCV infection must be assessed by a hepatologist for evidence of chronic liver disease. The following clinical situations should warrant a liver biopsy (AIII):

1. Associated iron overload.
2. History of excessive alcohol intake.
3. H/O hepatitis C for over 10–15 years.
4. Clinical evidence of chronic liver disease.

Patients with evidence of cirrhosis or hepatic fibrosis should not be considered for conventional conditioning (DIII).

### Risk of VOD:

Two studies have suggested that the risk of VOD is increased in patients with HCV infection and raised ALT pre-transplant, whilst others have suggested to the contrary. However, in patients with iron overload with HCV infection with raised ALT, VOD prophylaxis could be recommended routinely, in the absence of any conclusive evidence to the contrary (BIII).

### HSCT Donor:

Donors who are HCV-RNA positive invariably transmit HCV to uninfected recipient. Recipients show evidence of viremia in the immediate post-transplant period^42^. As mentioned above, the risks of short-term morbidity and mortality are extremely low. However, the effect on long-term survivors is significant. Donors who are anti-HCV positive but HCV-RNA negative, are unlikely to infect the recipient^42^. Thus, patients who desperately need a HSCT and do not have an alternative donor, can proceed with HSCT with full understanding of the long-term side-effects. The donor should be assessed for chronic liver disease and other extra-hepatic manifestations of HCV, which might contraindicate a bone marrow or stem cell harvest (EIII). All donors should be screened for ant-HCV antibodies. All anti-HCV positive donors should be tested for HCV-RNA. Those at high-risk of HCV infection should also be tested for HCV-RNA even if anti-HCV antibody is negative (AII).

## Preventing Progression of HCV-Associated Chronic Liver Disease

### Treating HSCT recipients:

The standard treatment for chronic hepatitis C infection is a combination of interferon and ribavirin, which produces 80% sustained virological response in genotypes 2 and 3 and 40–50% in genotype 1^43^,^44^. More recently, peginterferon 1.5 μg/kg once a week along with ribavirin was found to be more efficacious than standard IFN 3mU thrice a week^45^. Treatment for hepatitis C has not been considered seriously in HSCT recipients until recently, for several reasons. Firstly, the risk of early progression to cirrhosis was underestimated. Secondly, there were concerns regarding safety of IFN in HSCT recipients. A study on 36 HCV positive HSCT recipients treated with IFN alone or IFN plus ribavirin, reported a response rate of 10% and 20% respectively^39^. The major side-effects were related to anemia which was responsive to erythropoietin and thrombocytopenia, which necessitated interruption of treatment. The response rate was much lower than those observed in immunocompetent patients, but similar to HIV-coinfected population^46^.

Based on the current evidences, the following can be recommended (BIII):

1. Treatment for chronic HCV should be considered in all HSCT recipients in collaboration with a hepatologist. The indications are listed in Table 7.
2. Treatment should be initiated with full-dose peginterferon and ribavirin.
3. Dose modifications should be made based on tolerance and cytopenias
4. The treatment should be continued for 24–48 weeks depending on response.
5. Sustained Virological Response (SVR) is defined as negative HCV-RNA 24 weeks from completion of treatment

Limited data suggests improved outcome in HSCT recipients infected with HCV who respond to combination therapy.

### Preventing transmission of HCV from HSCT donor:

As mentioned above, HCV-RNA positive donors universally transmit virus to the recipient immediately following stem cell infusion. Donors who are anti-HCV positive but HCV-RNA negative are unlikely to infect the recipient. Based on these premises, it might be logical to attempt viral clearance in the donor prior to stem cell harvest, if feasible. There are few reports on success of this method^47^. Both the donor and recipient should be counselled on individual risks and the donor be treated with standard combination therapy (BIII).

## Conclusion:

Hepatitis viruses B and C are widely prevalent in the general population and affect the outcome of HSCT. Prevention is the key to overcoming problems related to these viruses. However, proper strategies adopted in transplanting infected patients or grafts from infected donors can minimise both short- and long-term complications and fatal outcomes.

## Figures and Tables

**Table 1.** Evidence-based rating system used in this article.

**Strength of Recommendation**	
**Category**	**Definition**
**A**	Both strong evidence for efficacy and substantial clinical benefit support recommendation for use. **Should always be offered**
**B**	Moderate evidence for efficacy—or strong evidence for efficacy, but only limited clinical benefit—supports recommendation for use. **Should generally be offered.**
**C**	Evidence for efficacy is insufficient to support a recommendation for or against use, or evidence for efficacy might not outweigh adverse consequences, (e.g., drug toxicity, drug interactions), or cost of the chemoprophylaxis or alternative approaches. **Optional**.
**D**	Moderate evidence for lack of efficacy or for adverse outcome supports a recommendation against use. **Should generally not be offered**.
**E**	Good evidence for lack of efficacy or for adverse outcome supports a recommendation against use. **Should never be offered**.

**Quality of Evidence supporting the recommendation**	
**Category**	**Definition**
**I**	Evidence from at least one well-executed randomized, controlled trial
**II**	Evidence from at least one well-designed clinical trial without randomization; cohort or case-controlled analytic studies (preferably from more than one center); multiple time-series studies; or dramatic results from uncontrolled experiments
**III**	Evidence from opinions of respected authorities based on clinical experience, descriptive studies, or reports of expert committees

**Table 2.** Definitions for hepatitis B infected states.

Chronic asymptomatic HBsAg carrier	HBsAg positive beyond 6 months with normal levels of liver enzymes (ALT and AST). Low level viral replication in serum.
Chronic symptomatic hepatitis B	As above with 3- fold raised liver enzymes (ALT and AST) on two separate occasions.
Occult HBV carrier	HBsAg negative, anti-HBc positive, anti-HBs positive or negative, low level viral replication in serum, normal liver enzymes.
Resolved HBV	History of proven hepatitis B infection with negative HBsAG and HBV DNA, normal liver enzymes, but positive anti-HBc and anti-HBs (may be negative)
HBV reactivation	symptomatic or asymptomatic rise in HBV DNA followed by rise in liver enzymes (ALT and AST) in a known HBsAg carrier.
Reverse sero-conversion	progressive loss of anti-HBs antibodies along with rising viral load and reappearance of HBsAg

**Table 3.** Investigations based on HBV sero-status of the recipient.

**HSCT RECIPIENT SERO-STATUS**	**INVESTIGATIONS**
HBsAg negative antiHBc positive anti-HBs positive	Repeat the profile If anti-HBs positive: proceed with HSCT If anti-HBs negative: check for HBV DNA If negative, complete vaccination schedule If HBV DNA positive: quantitate viral load and proceed with preemptive therapy (see text)
HBsAg negative antiHBc positive anti-HBs negative	Check HBV DNA If HBV DNA negative, complete vaccination schedule If HBV DNA positive, proceed as below.
HBsAg positive	Check viral load Proceed with pre-emptive therapy (see text)

**Table 4.** Investigatons for the donor based on donor and recipient sero-status.

**DONOR STATUS**	**RECIPIENT STATUS**	**RECOMMENDATIONS**
HBsAg negative anti-HBc positive anti-HBs negative	HBV-naïve	Check for HBV DNA If negative, check at the time of harvest Proceed without intervention, if negative Treat as Table 5 if positive
HBsAg negative anti-HBc positive anti-HBs positive	HBV-naïve	Confirm profile If antiHBs positive, proceed without intervention. If antiHBs negative, check HBV DNA and proceed accordingly.
HBsAg negative anti-HBc positive anti-HBs positive	HBsAg positive	Confirm profile Preferred donor compared to a HBV naïve donor (see text)

**Table 5.** Recommendations for transplant from HBsAg positive donor to HBV naïve recipient.

Measure HBV DNA in the patient. Exclude precore mutant form Treat donor with lamivudine for at least 4 weeks or until HBV DNA is undetectable (if time permits) Active immunisation of the patient with at least two doses at 4 weeks interval (poor response anticipated in severely pre-treated patients). Reduce harvest volume to minimum without compromising planned CD34positive cell dose. Test harvest product for HBV DNA If antiHBs titre is less than 10 IU/l, passive immunization with HBV immunoglobulin immediate pre-infusion of stem cells. If donor and harvest HBV-DNA negative and patient anti-HBs titre protective, consider monitoring with frequent HBV DNA and ant-HBs titre post-transplant and treat with lamivudine at the earliest sign of serconversion or detection of viral load. If donor or harvest positive for HBV-DNA, treat pre-emptively with lamivudine as above. Immunise recipient after immune recovery with 1–3 doses of HBV vaccine as needed, if remains uninfected (negative for HBsAg, anti-HBc, anti-HBs and HBV DNA). In addition, all HBsAg positive or HBV DNA positive donors should be evaluated for chronic hepatitis/cirrhosis and HCC prior to stem cell donation.

**Table 6.** **S**uggested management for patients at risk of HBV reactivation post-transplant.

Investigations	Liver function test: weekly for first 6 months then fortnightly until stoppage of all immunosuppressive and anti-viral therapy HBV-DNA: quantitative assay fortnightly if patient is not on anti-viral therapy monthly if on anti-viral therapy. Immediately if there is threefold rise in liver enzymes Fortnightly for three months after cessation of antiviral therapy If there is evidence of reverse sero-conversion at any stage
Management	Low risk: Monitoring and anti-viral therapy on earliest sign of HBV reactivation or reverse seroconversion. High-risk Prohylaxis with antiviral drugs until three months after cessation of all immunosuppressive medications, if there is no evidence of rising viral load. Change to second line antiviral therapy if there is a rise in viral load on first line therapy

**Table 7.** Indications for treatment of HCV post-transplant.

In CR from the original disease Two years post-transplant without evidence of chronic GVHD Off immunosuppression for 6 months Normal blood counts and serum creatinine Should have a baseline liver biopsy Be treated for iron overload if present, before initiating therapy No problems related to excess alcohol consumption
